# Author Correction: Waveguide-integrated twisted bilayer graphene photodetectors

**DOI:** 10.1038/s41467-024-49561-x

**Published:** 2024-06-21

**Authors:** Qinci Wu, Jun Qian, Yuechen Wang, Luwen Xing, Ziyi Wei, Xin Gao, Yurui Li, Zhongfan Liu, Hongtao Liu, Haowen Shu, Jianbo Yin, Xingjun Wang, Hailin Peng

**Affiliations:** 1 Center for Nanochemistry, Beijing Science and Engineering Center for Nanocarbons, Beijing National Laboratory for Molecular Sciences, College of Chemistry and Molecular Engineering, Peking University, 100871 Beijing, P. R. China; 2 Beijing Graphene Institute, 100095 Beijing, P. R. China; 3 Academy for Advanced Interdisciplinary Studies, Peking University, 100871 Beijing, P. R. China; 4 State Key Laboratory of Advanced Optical Communications System and Networks, School of Electronics, Peking University, 100871 Beijing, P. R. China; 5 School of Engineering, Peking University, 100871 Beijing, P. R. China

**Keywords:** Optical properties and devices, Optical properties and devices, Optoelectronic devices and components

Correction to: *Nature Communications* 10.1038/s41467-024-47925-x, published online 1 May 2024

The original version of this Article contained an error in the Methods section. The chemical formula (NH_4_)_2_S_2_O_8_ was erroneously written as (NH)_4_S_2_O_8_. This has been corrected in the PDF and HTML versions of the Article.

