# Supplementary material for: Waveguide-integrated twisted bilayer graphene photodetectors
Source: Nat Commun. 2024 May 1;15:3688. doi: 10.1038/s41467-024-47925-x (PMC11063206; doi:10.1038/s41467-024-47925-x)
Supplement: Supplementary file 1 — Supplementary Information [file 41467_2024_47925_MOESM1_ESM.pdf]

Supplementary information

Waveguide-integrated twisted bilayer graphene photodetectors

Qinci Wu[1,2†], Jun Qian[1,2†], Yuechen Wang[1,2,3†], Luwen Xing[4,5†], Ziyi Wei[4], Xin Gao[1,2,3], Yurui Li[2,3], Zhongfan Liu[1,2,3], Hongtao Liu[1], Haowen Shu[4], Jianbo Yin[2,3,4*], Xingjun Wang[4*], Hailin Peng[1,2,3*]

[1]*Center for Nanochemistry, Beijing Science and Engineering Center for Nanocarbons, Beijing National Laboratory for Molecular Sciences, College of Chemistry and Molecular Engineering, Peking University, Beijing 100871, P. R. China.*

[2]*Beijing Graphene Institute, Beijing 100095, P. R. China.*

[3]*Academy for Advanced Interdisciplinary Studies, Peking University, Beijing 100871, P. R. China.*

[4]*State Key Laboratory of Advanced Optical Communications System and Networks, Peking University, Beijing 100871, P. R. China.*

[5]*School of Engineering, Peking University, Beijing 100871, P. R. China.*

[†]These authors contributed equally to this work.

[*]Corresponding authors. E-mail: yinjb-cnc@pku.edu.cn, xjwang@pku.edu.cn, hlpeng@pku.edu.cn.

This file includes:

1. Supplementary Note 1. The optical conductivity and effective dielectric constant of graphene.
2. Supplementary Note 2. Mode analysis of waveguide-integrated graphene photodetectors.
3. Supplementary Note 3. Enhanced optical absorption of 4.1° tBLG for at 1,550 nm under surface light illumination.
4. Supplementary Note 4. High carrier mobility and contact resistivity of a field-effect transistor (FET) with tBLG as the active material.
5. Supplementary Note 5. The mechanisms of the waveguide-integrated tBLG photodetectors.
6. Supplementary Note 6. The power dependence of the waveguide-integrated graphene photodetectors.
7. Supplementary Note 7. The noise analysis of for the waveguide-integrated photodetectors.
8. Supplementary Note 8. The impendence analysis for the waveguide-integrated tBLG photodetectors.
9. Detailed information of the finite element simulations, fabrication processes, and performance evaluations for the waveguide-integrated photodetectors. (Supplementary Figs. 1–16).
10. Supplementary Table 1 | Comprehensive comparisons of the key figures of merit (FoMs) of waveguide-integrated graphene PDs in literature reports and our results.

Supplementary Note 1. The optical conductivity and effective dielectric constant of graphene.

A finite element method mode-solver (COMSOL Multiphysics) was used for the mode analysis of the waveguide-integrated graphene photodetectors (PDs). Graphene is incorporated by an equivalent volume permittivity model. The optical conductivity of single-layer graphene (SLG) was calculated from the Kubo formula, given by:

$$\sigma_{\mathrm{SLG}} = \frac{\sigma_0}{2}\left[\tanh\left(\frac{\hbar\omega+2\mu}{4k_BT}\right) + \tanh\left(\frac{\hbar\omega-2\mu}{4k_BT}\right)\right], \quad \text{(S1)}$$

where the $\sigma_0 = e^2/4\hbar$ is the universal optical conductivity of SLG under the linear-band regime, ω is the incident optical frequency, μ and T are the chemical potential and the temperature of SLG, respectively[1].

In the simulations, the thickness of SLG h_G was set to 0.34 nm. The bulk conductivity of SLG is expressed as σ/h_G, and the effective dielectric constant of SLG $\varepsilon_G(\omega)$ can be described as Equation (S2):

$$\varepsilon_G(\omega) = 1 + \frac{i\sigma(\omega)}{\omega\varepsilon_0 h_G}, \quad \text{(S2)}$$

where ε_0 is the vacuum permittivity[2]. $\varepsilon_G(\omega)$ can be expressed by tensor form:

$$\widehat{\varepsilon_G}(\omega) = \begin{bmatrix} n_C & 0 & 0 \\ 0 & \varepsilon_G & 0 \\ 0 & 0 & \varepsilon_G \end{bmatrix}. \quad \text{(S3)}$$

The horizontal component of effective dielectric constant $\varepsilon_G(\omega)$ can be obtained by equation (S2), and the vertical component is set as a constant $n_c = 2.5$[3,4].

Unlike the linear band structure of single layer graphene (SLG) and parabolic-like band structure of AB stacked bilayer graphene (BLG), the Dirac cones of the two individual monolayers in tBLG intersect and form saddle points in reciprocal space, resulting in the formation of van Hove singularities (vHs) in the density of state (DOS)[5,6]. In addition, the position of the intersection (and thus vHs) is θ-dependent. The energy difference between the vHs can be described as $\Delta E_{\mathrm{vHs}} = 2\hbar v_{\mathrm{F}} K sin(\frac{\theta}{2})$. When the incident photon energy matches ΔE_{vHs}, a pronounced interband transition between vHs and an enhanced absorption happen [3]. This can be understood by the joint density of states (JDOS):

$$\mathrm{JDOS}(\omega) = \frac{1}{4\pi^3}\int \delta[E_c(\mathbf{k}) - E_v(\mathbf{k}) - \hbar\omega]d\mathbf{k} \quad \text{(S4)}$$

Where E_c, E_v, $\mathbf{k}$ and $\hbar$ are conduction band energy, valance band energy, reciprocal vector, and photon energy, respectively. The integration indicates that the JDOS is highly relevant to the density of states (DOS). For tBLG, when these two singularities match the photon energy, which means the interband transition matches the delt function in the equation R1, the absorption is enhanced. For example, the photon energy of 0.8 eV corresponds to an incident λ of 1,550 nm, θ is thus estimated to be ~4.1°.

The enhanced optical absorption of tBLG can be calculated based on its optical conductivity σ_{tBLG}, which comes from two distinct interband transitions[7]: the linear-band transition σ_{SLG} of SLG and the transition σ_α from the van Hove singularities (vHs) to the band edges (BEs), as described by

$$\sigma_{\mathrm{tBLG}} = 2\sigma_{\mathrm{SLG}} + \sigma_\alpha. \quad \text{(S5)}$$

σ_α can be described by the standard Gaussian function:

$$\sigma_\alpha = \frac{S_\alpha}{\sqrt{2\pi\Gamma_\alpha^2}} \exp\left[-\frac{(\hbar\omega - E_\alpha)^2}{2\Gamma_\alpha^2}\right], \quad \text{(S6)}$$

where S_α, E_α, and Γ_α represent the intensity, energy, and broadening of the optical conductivity peak, respectively. The twist angle-dependent optical conductivity of tBLG was calculated using function (S5), as shown in Fig. 1c. According to the previous theoretical calculation and experiment demonstrations[7,8], the optical conductivity of tBLG can reach up to $3\sigma_{SLG}$ when the energy difference of two vHs in tBLG matches the incident wavelength, different from that of AB-stacked BLG ($2\sigma_{SLG}$). As illustrated in Fig. 1c, σ_{tBLG} exhibits a remarkable peak at a twist angle of ~4.1° corresponding to an incident light wavelength of 1,550 nm, and retains greater than $2\sigma_{SLG}$, when the θ deviation is within 1°. These calculation results thus indicate that tBLG exhibits larger optical conductivity mainly responsible for enhanced absorption.

Supplementary Note 2. Mode analysis of waveguide-integrated graphene photodetectors.

The absorption enhancement of tBLG is compatible to absorption of field enhancement from plasmonic effect[6], which means these two enhancement factors can be multiplied to give rise larger enhancement. In principle these two processes are independent: The field enhancement can be seen as "concentration of optical field (or optical beam)" as the light intensity equals the square of the optical electric field". It happens at the optical illuminating process. On the other hand, the absorption enhancement of tBLG happens at the electron excitation process and is due to the enhanced joint density of states (or van Hove singularities).

For a given propagation length of L, the graphene absorptance $\eta(L)$ can be expressed by

$$\eta(L) = \eta_{\mathrm{G}}(1 - 10^{-0.1\alpha L}), \quad \text{(S7)}$$

where η_G is the light absorption ratio of graphene, and $\alpha = \alpha_G + \alpha_M$ is the total absorption coefficient in dB μm^{-1}. The light absorption ratio η_G is given by

$$\eta_{\mathrm{G}} = \frac{\alpha_{\mathrm{G}}}{\alpha} = \frac{\alpha_{\mathrm{G}}}{\alpha_{\mathrm{G}}+\alpha_{\mathrm{M}}}, \quad \text{(S8)}$$

The graphene absorptance is calculated by[9]

$$\eta(L) = \frac{\int_0^L \int A_{\mathrm{G}}(l) e^{-\alpha_{\mathrm{e}} z} \mathrm{d}l\mathrm{d}z}{P_0} = \frac{1}{\alpha_{\mathrm{e}}}(1 - e^{-\alpha_{\mathrm{e}} L}) \frac{\int A_{\mathrm{G}}(l)\mathrm{d}l}{P_0}, \quad \text{(S9)}$$

where P_0 is the incident power, l is the coordinate of the line integral along the graphene surface in the xy plane, $\alpha_e = \alpha/4.34$ is the mode absorption coefficient in μm^{-1}, and $A_G(l)$ is the light absorption intensity in W/m^2 of graphene. $A_G(l)$ is given by

$$A_{\mathrm{G}}(l) = \frac{1}{2}\sigma_{\mathrm{G}} |\mathbf{E}_{\mathrm{t}}(l)|^2, \quad \text{(S10)}$$

where $\mathbf{E}_t$ is the transverse component of the electric fields along the graphene surface of the launched waveguide mode. Similarly, the metal absorptance $\eta_M(L)$ is calculated by

$$\eta_{\mathrm{M}}(L) = \frac{\int_0^L \iint A_{\mathrm{M}}(x,y) e^{-\alpha_{\mathrm{e}} z} \mathrm{d}x\mathrm{d}y\mathrm{d}z}{P_0} = \frac{1}{\alpha_{\mathrm{e}}}(1 - e^{-\alpha_{\mathrm{e}} L}) \frac{\iint A_{\mathrm{M}}(x,y)\mathrm{d}x\mathrm{d}y}{P_0}. \quad \text{(S11)}$$

Here, the integral area of the xy-plane surface integral is the metal area, and $A_M(x, y)$ is the light absorption intensity (W/m^3) of metal, given by

$$A_{\mathrm{M}}(x,y) = \frac{1}{2}\omega \cdot \varepsilon_{\mathrm{M}\_}im(\omega) |\mathbf{E}(x,y)|^2, \quad \text{(S12)}$$

where $\varepsilon_{M\_}im(\omega)$ is the imaginary part of the metal permittivity, and $\mathbf{E}$(x, y) is the electric fields in metal. According to Equation (S8) and (S10), the graphene absorption coefficient α_G and the metal absorption coefficient α_M are given as (in dB μm^{-1})

$$\alpha_{\mathrm{G}} = 4.34 \frac{\int A_{\mathrm{g}}(l) dl}{P_0}, \quad \text{(S13)}$$

$$\alpha_{\mathrm{M}} = 4.34 \frac{\iint A_{\mathrm{m}}(x,y) dx dy}{P_0}, \quad \text{(S14)}$$

With Equation (S12) and (S13), the absorption coefficients α_G, α_M and the graphene absorption ratio η_G can be calculated. The simulation results of the waveguide-integrated graphene PDs operating at 1,550 nm are shown in Supplementary Fig. 3. The optical conductivities and the effective dielectric constant of SLG, AB-stacked BLG, and 4.1° tBLG are given in Supplementary Note 1. As the metal width W_m increases, the metal absorption coefficient α_M grows rapidly, while α_G saturates when $W_m > 200$ nm. Therefore, the metal width W_m is set as 200 nm. In this case, (α_G, η_G) of SLG, AB-stacked bilayer graphene (BLG), and 4.1° tBLG are calculated to be (0.184 dB μm^{-1}, 52.9%), (0.394 dB μm^{-1}, 70.8%), and (0.577 dB μm^{-1}, 78.8%), respectively. The

calculated graphene absorptance $\eta(L)$ varying with the propagation length L is plotted in Fig. 1d in the main text.

Supplementary Note 3. Enhanced optical absorption of 4.1° tBLG at 1,550 nm under surface light illumination.

Previous studies have corroborated the enhanced optical absorption of tBLG covering visible, near- and mid-infrared wavelengths under surface light illumination[6,7,10]. However, it has not been demonstrated for a standard telecom wavelength of 1,550 nm. To verify this, a photodetector was designed and fabricated on SiO_2/Si substrate with a channel simultaneously comprising a SLG domain and a 4.1° tBLG domain. The spatially resolved photocurrent mapping of the device was characterized by scanning photocurrent microscopy. The junction of metal/4.1° tBLG domain generates significantly larger photocurrent (~4 times) than that of the SLG ones at zero bias under 1,550 nm direct illumination (see Supplementary Fig. 6), revealing an enhanced optical absorption of 4.1° tBLG at 1,550 nm. This result also suggests the feasibility of 4.1° tBLG for the construction of waveguide-integrated photodetector demonstrating high photoresponsivity.

Supplementary Note 4. Carrier mobility and contact resistivity of a field-effect transistor (FET) with tBLG as the active material.

Four-probe electrical measurements were used to extract the carrier mobility and the contact resistivity of a tBLG FET. Two probes were used to apply a source-drain voltage V_{DS} and measure the source-drain current I_{DS}, and the other two probes were used to measure the voltage drop along the graphene channel between V_1 and V_2 (see Fig. 2c in the main text). A global gate was introduced by fabricating the FET on a highly doped silicon substrate capped with a 300-nm thick SiO_2 as gate dielectric. Gate voltage V_G was swept from 60 to −60 V. The resistance of the whole channel, R_{DS}, and the channel region between probe 1 and 2, R_{12} can be expressed as follows:

$$R_{DS} = \frac{V_{DS}}{I_{DS}}, \tag{S15}$$

$$R_{12} = \frac{V_1 - V_2}{I_{DS}}. \tag{S16}$$

The carrier mobility μ can be calculated by:

$$\mu = \frac{\partial(\frac{I_{DS}}{V_1 - V_2})}{\partial V_G} \cdot \frac{L_{12}}{W} \cdot \frac{1}{C_{OX}}, \tag{S17}$$

where L_{12} is the length between probe 1 and 2, W is the channel width, and C_{OX} is the gate insulator capacitance per unit area. The contact resistivity R_C is given by:

$$R_C = (R_{DS} - R_{12}\frac{L_{DS}}{L_{12}}) \cdot \frac{W}{2}, \tag{S18}$$

where L_{DS} is the channel length. With these formulas, the hole (electron) mobility μ of 10,600 (9,100) $cm^2\ V^{-1}\ s^{-1}$ was obtained, and the contact resistivity in this device was estimated to be ~500 Ω cm (see Supplementary Fig. 7). Additionally, given that the contact resistance can be expressed as $2R_C/W$, where R_C is the contact resistivity, and W is tBLG length. Therefore, the contact resistance in our tBLG photodetector is ~125 Ω considering our tBLG length is 8 μm.

Supplementary Note 5. The mechanisms of the waveguide-integrated tBLG photodetectors.

The mechanisms of graphene photodetection include photo-thermoelectric (PTE), photoconductive (PC) and photo-bolometric (PB) effects. PTE effect origins from diffusivity (Seebeck coefficient) differences of hot electrons at different doping levels[11]. It dominates when the source-drain bias is zero or small. In PTE effect, the photocurrent polarity (flowing direction) shouldn't change with source-drain bias polarity. PC effect origins from the migration of hot electrons under source-drain bias, which dominates at relatively large source-drain bias[12,13]. In this regime, the polarity of photocurrent should be the same with that of the source-drain bias. As we have explained in the last response, the PB effect origins from resistivity differences at different lattice temperature[12,13]. In this regime, light illumination heats the lattice, increase the resistivity, and decrease the total current, so the "photocurrent', defined as difference of current with and without illumination, should show opposite polarity with the source-drain bias. Therefore, with the polarity of photocurrent as key evidence[11] and the same polarity of photocurrent with source-drain bias in Supplementary Fig. 10, We conclude that the PC effect is the dominating mechanism in our devices.

The device performance is almost symmetric under positive and negative bias voltages in Supplementary Fig. 11 because the bias voltages act as photocurrent extracting "forces" and should extract similar scale but opposite polarities. The other notable feature is the nonlinear dependence of photocurrent (responsivity) with bias voltage. This is due to that with voltage increasing the extracting efficiency increases and the main bottle neck lies on the photocurrent numbers which is mainly dependent on incident power[11].

Supplementary Note 6. The power dependence of the waveguide-integrated graphene photodetectors.

We have normalized the responsivities shown in Fig. 2d and show the relations in Supplementary Fig. 13. It seems that these three curves have similar trends, implying that the seemingly stronger power dependence of tBLG responsivity should mainly come from its high baseline value (higher responsivity values at each point). In fact, this dependence trend has been observed before[9,12,14–16] and ascribed to the sublinear electron heating response under illumination[17]. tBLG has higher absorption, which gives rise to higher electron temperature (can be seen as calibration of the photocarrier density). Therefore, in principle, tBLG should have slightly stronger power dependence due to the sublinear electron heating. This might explain the slightly stronger dependence as shown by red curve in Supplementary Fig. 13.

Supplementary Note 7. The noise analysis for the waveguide-integrated photodetectors.

To evaluate the sensitivity performance of the device, we calculated the frequency-normalized noise equivalent power (NEP), i.e., the amount of incident light power that generates a photocurrent equal to the noise current (NEP = i_n/R_{ph}, where i_n is noise current and R_{ph} is the photoresponsivity). The noise current mainly consists of the dark current shot noise current $\sqrt{\overline{i_{nd}^2}}$ and the Johnson current noise (thermal noise) current $\sqrt{\overline{i_{nJ}^2}}$, which are given by

$$\sqrt{\overline{i_{nd}^2}} = \sqrt{2qI_d}, \tag{S19}$$

$$\sqrt{\overline{i_{nJ}^2}} = \sqrt{\frac{4k_BT}{R_{tot}}}, \tag{S20}$$

where I_d is the dark current, R_{tot} is the total device resistance, q is the unit charge, k_B is the Boltzmann constant, and $T = 300$ K is the operating temperature. NEP is given by

$$\mathrm{NEP} = \frac{i_n}{R_{ph}} = \frac{\sqrt{\overline{i_{nd}^2}+\overline{i_{nJ}^2}}}{R_{ph}} = \frac{\sqrt{2qV_b+4k_BT}}{R_{ph}\sqrt{R_{tot}}}. \tag{S21}$$

The extracted NEP and noise current are plotted in Supplementary Fig. 12. The NEP of tBLG device is found to be 46–98 pW $Hz^{-1/2}$, which is greatly lower than that of SLG (187–349 pW $Hz^{-1/2}$) and AB-stacked BLG ones (90–196 pW $Hz^{-1/2}$).

Supplementary Note 8. The impendence analysis for the waveguide-integrated tBLG photodetectors.

To estimate the limiting factor for the 3 dB bandwidth f_{3dB} in the waveguide-integrated tBLG photodetector in the main text, an equivalent electrical circuit model of the device was established, as shown in Supplementary Fig. 16. f_{3dB} is determined by both RC-limited bandwidth f_{RC} and transit-time-limited bandwidth f_{tr},

$$f_{3dB} = (\frac{1}{f_{RC}^2} + \frac{1}{f_{tr}^2})^{-1/2}. \quad \text{(S22)}$$

The transit-time-limited bandwidth f_{tr} can be calculated according to the following equation:

$$f_{tr} = \frac{3.5}{2\pi t_{tr}}, \quad \text{(S23)}$$

where $t_{tr} = W^2/\mu V_b$ is the carrier transit time through the photodetection region, and W is the source–drain separation distance. Thus, a value of f_{tr} = 295 GHz is calculated with W = 1 μm and μ = 10,600 cm^2 V^{-1} s^{-1}. On the other hand, the RC-limited bandwidth f_{RC} is given by:

$$f_{RC} = \frac{1}{2\pi R(C_{pad}+C_g)}, \quad \text{(S24)}$$

where C_{pad} and C_g are the capacitance of the metal pad and graphene, respectively, R includes the parasitic resistance and the load resistance R_L (50 Ω). The device resistance R_{tot} = 150 Ω can be extracted by the static I–V curves. The channel capacitance C_g and the pad capacitance C_{pad} are calculated to be 0.36 fF and 5.7 fF by the three-dimensional numerical simulation method (COMSOL), respectively. The f_{RC} is thus estimated to be 133 GHz. f_{3dB} can be expected to reach up to 121 GHz, which is limited mainly by RC constant. Therefore, in principle as long as the photocurrent signal is measurable and the RC value is unchanged, the bandwidth should be independent of source-drain bias. This can be evidenced by the similar bandwidths under different biases in Supplementary Fig. 15. The 3-dB bandwidth keeps at about 65 GHz under bias voltages of 0.2 V, 0.5 V and −0.5 V.

Detailed information of the finite element simulations, device fabrication processes, and performance evaluations for the waveguide-integrated photodetectors.

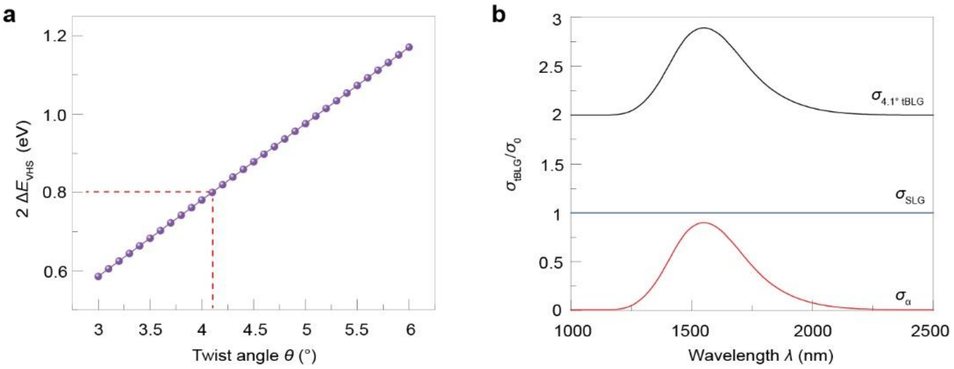

Supplementary Fig. 1 | Twist angle determination and enhanced optical conductivity of tBLG. a, Relationship between the energy interval of vHs and the twist angle. When $2\Delta E_{vHs}$ (~ 0.8 eV) matches an incident wavelength λ of 1,550 nm, the corresponding twist angle of tBLG is estimated to be ~4.1°. **b**, Optical conductivity σ_{tBLG} of 4.1° tBLG as a function of λ. σ_{tBLG} can be expressed as a sum of $2\sigma_{mono}$ and σ_{α}. The optical conductivity of 4.1° tBLG peaks at λ = 1,550 nm, which is the particular origin for the enhanced light-matter interaction.

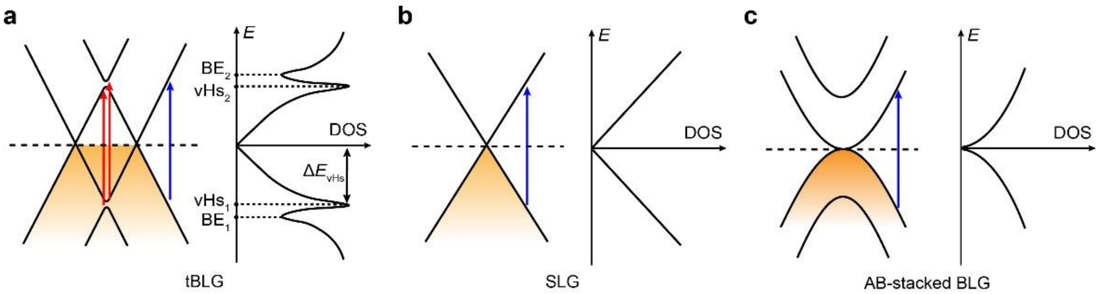

Supplementary Fig. 2 | Schematic illustration of band structure and optical transition of tBLG, SLG and AB-stacked BLG. a-c, Different optical transition in 4.1° tBLG (**a**), SLG (**b**), and AB-stacked BLG (**c**), respectively. blue rows represent the linear-band transition, and the red rows represent the transition between VHSs and BEs in tBLG.

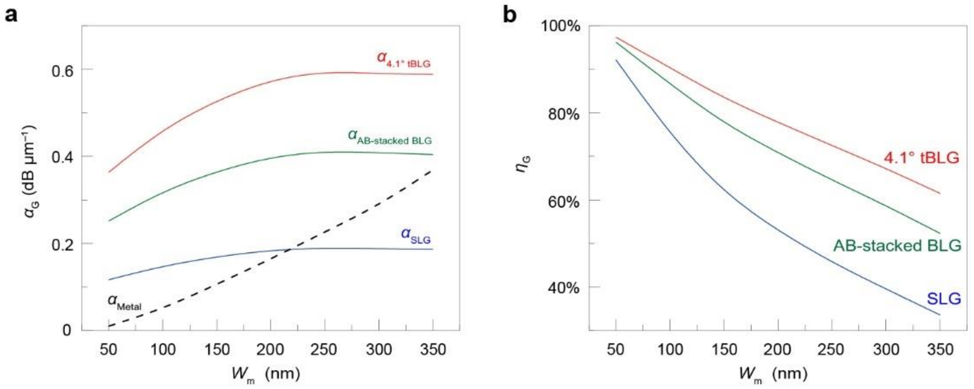

Supplementary Fig. 3 | Simulated absorption coefficients and graphene absorption ratio of the designed graphene photodetectors integrated with silicon waveguide (SiWG). a, Absorption coefficients α of the metal electrode α_{Metal} (dashed line) and the graphene α_{G} (solid line) as a function of the signal electrode width W_{m}. The calculations were performed at a λ of 1,550 nm wavelength with a transverse electric (TE) polarized mode. α_{Metal} increases with W_{m}, and α_{G} stays constant when $W_{\mathrm{m}} > 200$ nm. **b**, Calculated graphene absorption ratio η_{G} as a function of W_{m}.

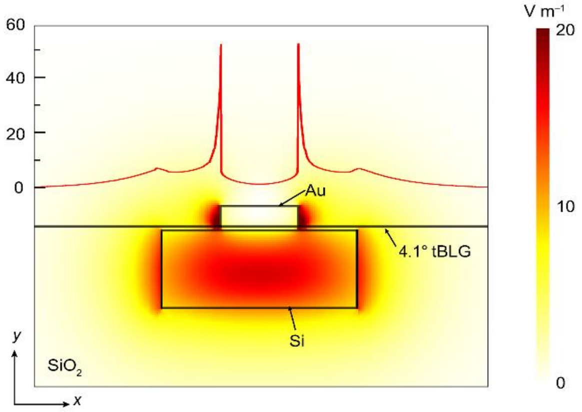

Supplementary Fig. 4 | Simulated electric-field profile of the TE waveguide mode. The field distribution along the tBLG sheet is shown as the red line. A strong field localization occurs around the metal/tBLG interface, which is greatly beneficial for the highly efficient photon absorption.

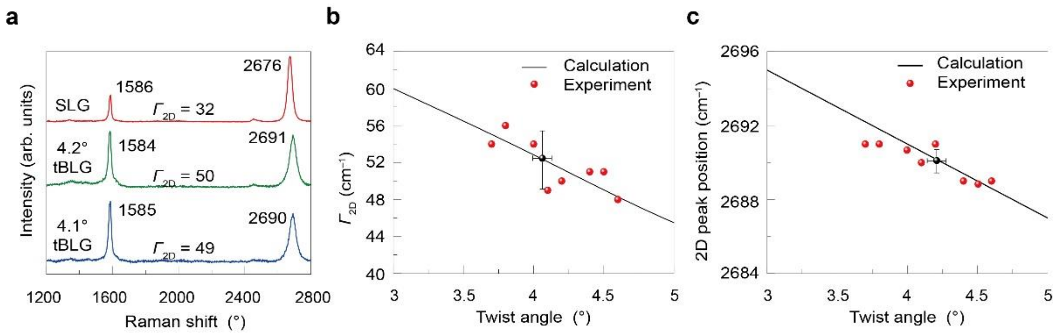

Supplementary Fig. 5 | Twist angle-dependence of Raman 2D peak. a, Raman spectra of SLG and tBLG measured with 633 nm wavelength laser. **b**, Raman 2D peak full-width at half maximum (Γ_{2D}) as a function of twist angle. **c**, Raman 2D peak position as a function of twist angle. Eight tBLG domains with ~4.1° twist angle were characterized by Raman spectrum with 633 nm wavelength laser. The broadening and blueshift of 2D band are attributed to the twist angle-dependence electronic band structure due to the interlayer interaction. The black line and red circles represent the theoretical calculation and experimental values, respectively. The measured twist angle of Raman 2D peak is thus in excellent agreement with the theoretical calculations[18].

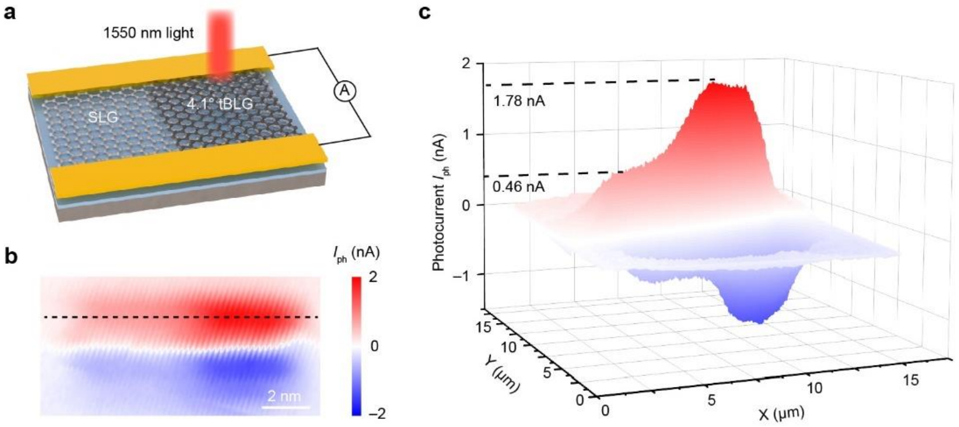

Supplementary Fig. 6 | Enhanced photocurrent generation in 4.1° tBLG. a, Schematic illustration of the photodetector on SiO_2/Si substrate. The channel comprises a 4.1° tBLG domain and a SLG domain. **b**, Scanning photocurrent image of the particular device. A 1,550-nm laser with the power of 10 μW was illuminated on the device. The photocurrent was amplified and recorded by a lock-in amplifier without source-drain bias. **c,** Three-dimensional view of the scanning photocurrent image of the device. The peak current corresponds to the black dashed line in (**b**).

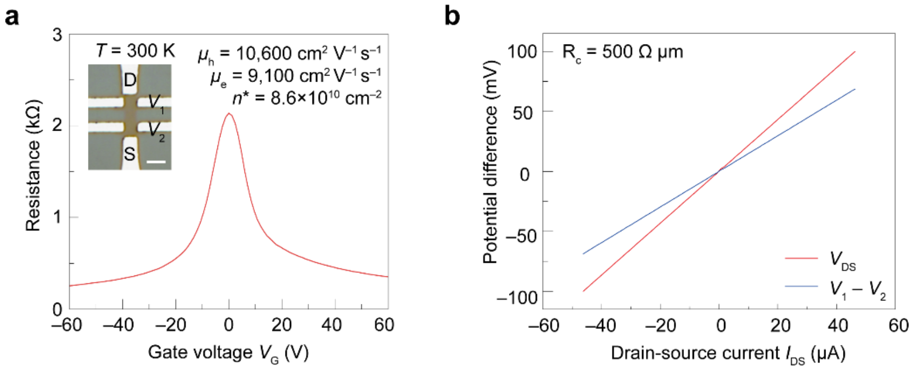

Supplementary Fig. 7 | Carrier mobility and contact resistivity of the tBLG FET. **a**, Four-probe resistance as a function of gate voltage for a tBLG back-gate field-effect transistor (FET) at room temperature. Inset: the OM image of the corresponding tBLG Hall bar device. Scale bar: 2 µm. **b**, The drain-source voltage V_{DS} and the voltage difference between V_1 and V_2 as a function of the drain-source current I_{DS} at $V_G = 0$ V. Contact resistivity was calculated to be 500 Ω µm using Equation (S17).

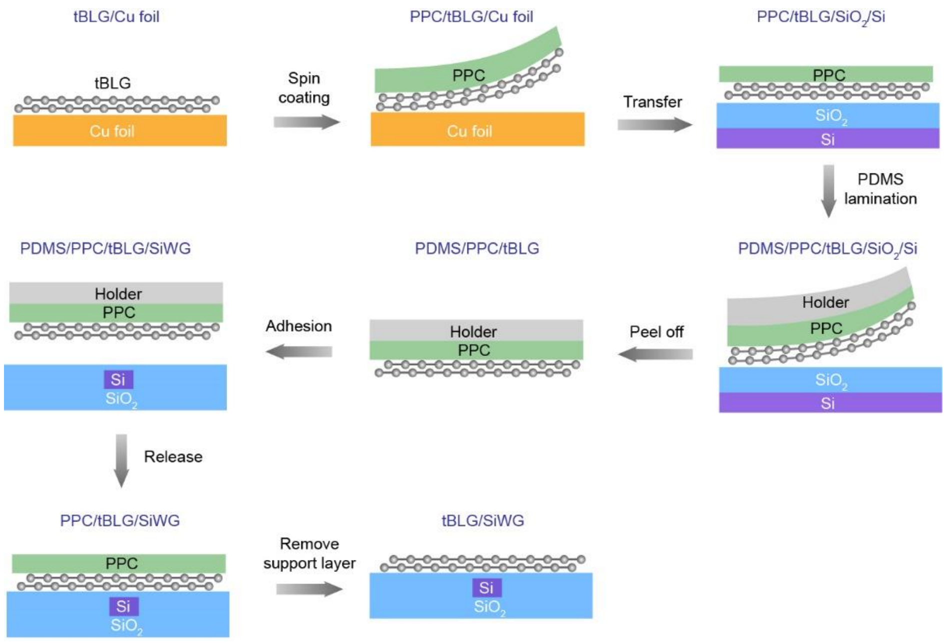

Supplementary Fig. 8 | Schematic of the process of transferring tBLG from Cu foil onto SiWG. tBLG was grown on Cu(111) foil by chemical vapor deposition (CVD). The samples with a desired twist angle of 4.1° and size were selected and marked under optical microscope. Then the selected samples were transferred using the PPC-assisted technique onto the SiO_2/Si substrate. A PDMS thin film was laminated onto the PPC film as a holder, and the PDMS/PPC/tBLG was detached from the SiO_2/Si substrate. Next, the PDMS/PPC/tBLG stamp was aligned and placed onto the SiWG by the deterministic transfer system. Finally, the PDMS was peeled off from the PPC film, and the PPC was dissolved in hot acetone leaving tBLG on SiWG.

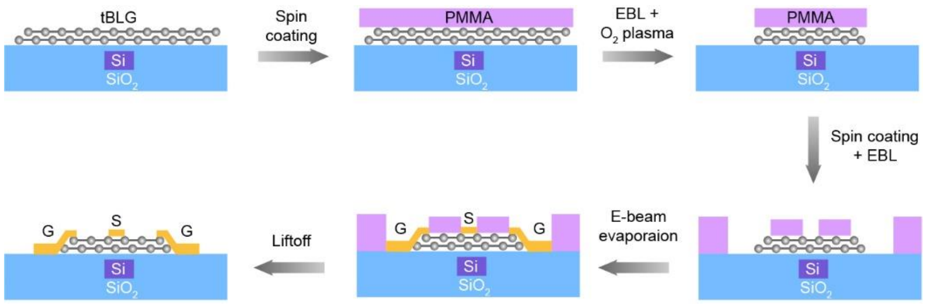

Supplementary Fig. 9 | Schematic of the process of fabrication of waveguide-integrated tBLG photodetectors. PMMA (950K, A4.5) was spin-coated on the as-transferred tBLG sample on the SiWG, followed by EBL and O_2 plasma etching to pattern tBLG into a channel geometry. After that, the tBLG/SiWG was spin-coated with MMA/PMMA, and the ground-signal-ground (GND-S-GND) regions of devices were defined by the same EBL process. Pd/Au (5/50 nm) were deposited by thermal evaporation, followed by a standard lift-off technique.

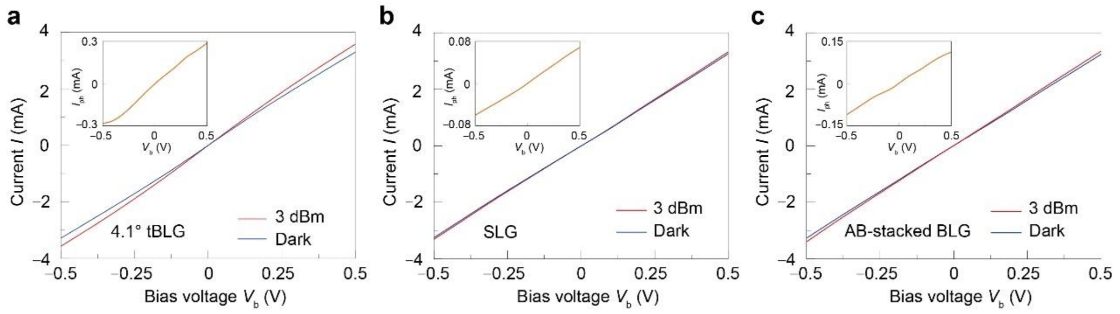

Supplementary Fig. 10 | Current-voltage characteristics of graphene PDs. a–c, I–V_b curves under dark and incident power of 3 dBm of 4.1° tBLG (**a**), SLG (**b**), and AB-stacked BLG (**c**) PDs, respectively. Inset: The corresponding I_{ph} of 4.1° tBLG (**a**), SLG (**b**), and AB-stacked BLG (**c**) PDs, respectively. Given a non-zero bias voltage, the predominant mechanisms are likely to be the PC effect or the photo-bolometric (PB) effect, with the photo-thermoelectric (PTE) effect being excluded. Considering that the polarity between the photocurrent and bias can be used to distinguish PC and PB effects[12]. We can see that, the current under light is larger than that in dark when applying external electric bias. Therefore, the same polarity between the photocurrent and bias is observed in all photodetectors using three kinds of graphene, indicating that the PC effect is the dominating photodetection mechanism in our devices.

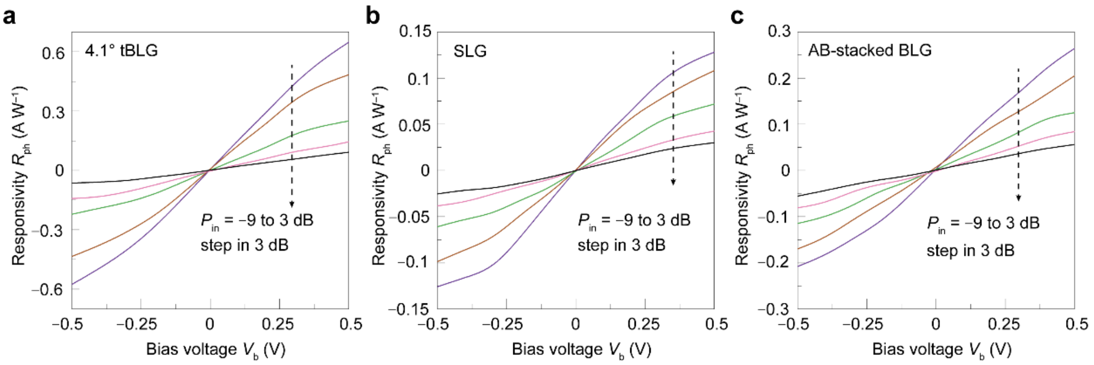

Supplementary Fig. 11 | Steady-state characterization of graphene PDs. a–c, R_{ph}–V_b curves at different incident power P_{in} of 4.1° tBLG (**a**), SLG (**b**), and AB-stacked BLG (**c**) PDs, respectively. The device performance is almost symmetric under positive and negative bias voltages.

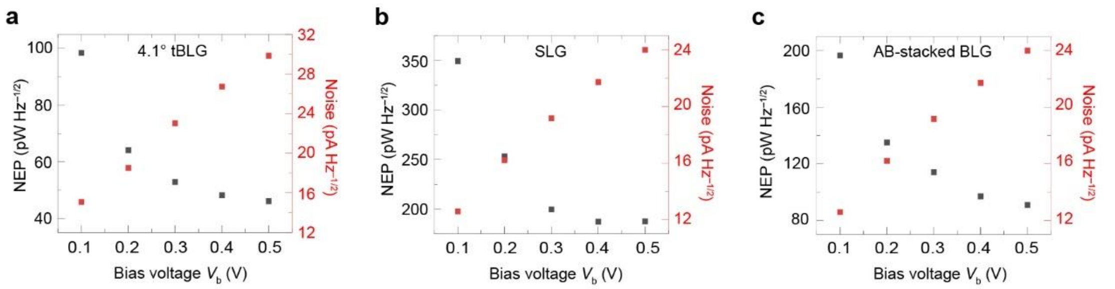

Supplementary Fig. 12 | Noise equivalent power (NEP) and noise current of graphene PDs. a–c, NEP (the black square) and noise current (the red square) as a function of bias voltage of 4.1° tBLG (**a**), SLG (**b**), and AB-stacked BLG (**c**) PDs, respectively.

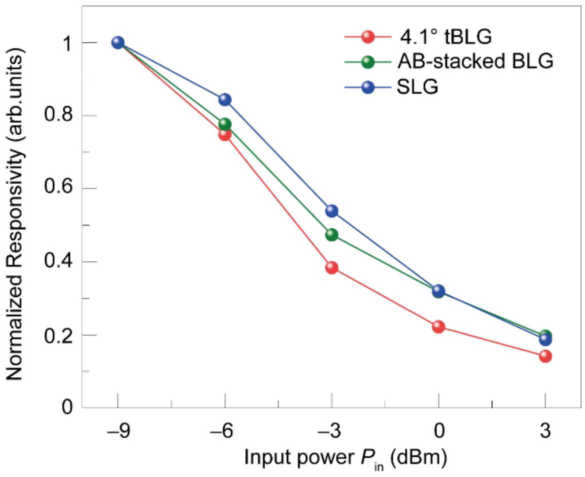

Supplementary Fig. 13 | Normalized power-dependent responsivities of different graphene devices as indicated by the legends. This figure uses the same data as those in Fig. 2d in the main text.

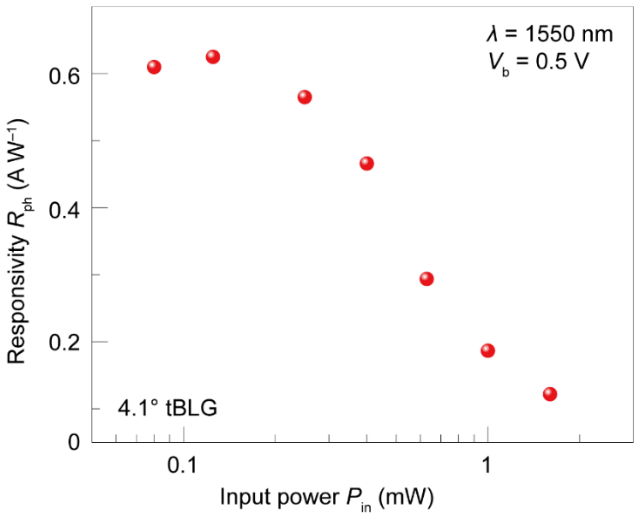

Supplementary Fig. 14 | Power dependence of the photoresponsivity at a V_b of 0.5 V for 4.1° tBLG photodetector. As the input power decreases to about –9 dBm (~0.125 mW), the responsivity attains the upper limit and remains constant even with further decreasing in the input power.

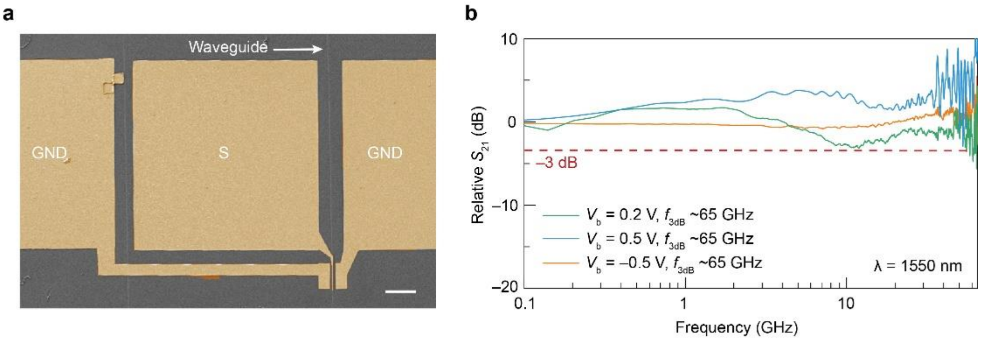

Supplementary Fig. 15 | High-frequency response of waveguide-integrated tBLG photodetectors. (a), Optical image of the device, scale bar: 10 μm. (b), Bandwidth data of the device in (a). Green, blue and orange lines denote measurement at bias voltages of 0.2 V, 0.5 V and −0.5 V, respectively. 3-dB cutoff is marked by the red dash line.

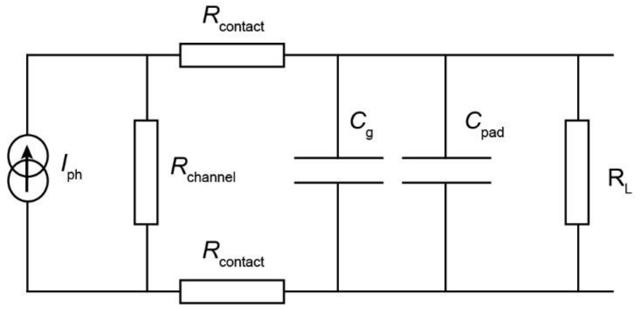

Supplementary Fig. 16 | The simplified equivalent electric circuit of the tBLG photodetector connected with a 50 Ω load (R_L).

Supplementary Table 1 | Comprehensive comparisons of the key figures of merit (FoMs) of waveguide-integrated graphene PDs in literature reports and our results.

Materials	Device structures (SiWG)	Operating wavelength (nm)	Dominant mechanisms	Photoresponsivity (mA W^{-1}) (V_b (V), P_{in} (mW))	3 dB bandwidth (GHz)	Data rate (Gbit s^{-1}, modulation format)	Energy consumption (pJ bit^{-1})	Ref.
Exfoliated-BLG	Planar diode (Si)	1,550	PC*	100 (1, 1)	20	12 (OOK)	NA	19
Exfoliated-BLG	GND-S-GND diode (Si)	1,550	PC	50 (0, 0.1)	18	NA	0.0005	20
Exfoliated-SLG	Vertical heterostructures (Si)	2,750	PV**	150 (1.5, 0.00094)	NA	NA	NA	21
CVD-SLG	Planar diode (Si)	1,550	PC	46 (0, 1)	41	50 (OOK)	NA	22
CVD-SLG	Phototransistor (Si)	1,550	PV	57 (0.4, 0.1)	3	NA	NA	23
CVD-SLG	Phototransistor (Si)	1,550	PTE***	360 (1.2, 0.0025)	42	NA	NA	24
CVD-SLG	Vertical heterostructures (Si)	1,550	PV	370 (3, 0.006)	NA	NA	NA	25
Exfoliated-SLG	Split-gate structure (Si)	1,550	PTE	35 (0, 0.8)	65	NA	NA	26
			PC	76 (0.3, 0.1)				
CVD-SLG	Planar diode (Si)	1,550	PB****	1 (1, 63)	76	12.5 (OOK)	NA	27
CVD-SLG	Planar diode (SiN)	1,550	PC	15 (0, 1.1)	30	NA	NA	28
CVD-SLG	Split-gate structure (Si)	1,550	PC	170 (0, 6.5)	40	NA	NA	29
CVD-SLG	Planar diode (Si)	1,550	PB (Plasmonic)	500 (0.4, 0.08)	110	100 (OOK)<br>100 (PAM4)	NA	15
CVD-SLG	Split-gate structure (SiN)	1,550	PTE (Plasmonic)	12.2 V W^{-1} (0, 0.1)	42	NA	NA	30

*PC: photoconductive effect; **PV: photovoltaic effect; ***PTE: photo-thermoelectric effect; ****PB: photobolometric effect.

Supplementary Table 1 (continued) | Comprehensive comparisons of the key figures of merit (FoMs) of waveguide-integrated graphene PDs in literature reports and our results.

Materials	Device structures (SiWG)	Operating wavelength (nm)	Dominant mechanisms	Photoresponsivity (mA W^{-1}) (V_b (V), P_{in} (mW))	3 dB bandwidth (GHz)	Data rate (Gbit s^{-1}, modulation format)	Energy consumption (pJ bit^{-1})	Ref.
CVD-SLG	Planar diode (Si)	1,550	PB (Plasmonic)	360 (1.9, 0.4)	110	40 (RZ)	NA	31
CVD-SLG	GND-S-GND phototransistor (Si)	1,550	PB (Plasmonic)	400 (–0.3, 0.1)	40	30 (OOK)	NA	9
		2,000	PB (Plasmonic)	70 (–0.3, 0.16)	20			
CVD-SLG	Vertical heterostructures (Si)	1,300	PV	200 (–3, 0.15)	24	NA	NA	32
CVD-SLG	Planar diode (Si)	1,550	PB	25 (0.3, 0.12)	17	NA	NA	33
CVD-SLG	Split-gate structure (SiN)	1,550	PTE	6 V W^{-1} (0, 1)	65	NA	NA	34
CVD-SLG	Split-gate structure (Si)	1,550	PTE	3.5 V W^{-1} (0, 1.65)	65	105 (OOK)<br>120 (PAM4)	NA	35
CVD-SLG	Split-gate structure (Si)	1,550	PTE	90 V W^{-1} (0, 0.6)	12	NA	NA	16
CVD-SLG	Planar diode (Si)	1,550	PC	100 (0.3, 5)	67	90 (BPSK)<br>200 (QPSK)<br>240 (16 QAM)	0.014	36
CVD-SLG	Vertical heterostructures (Si)	1,550	PV	0.15(–5, 1.86)	30	NA	NA	37
		1,960		0.015(–5, 1.96)				
CVD-SLG	Vertical heterostructures (Si)	1,550	PV	273(2, 0.5)	NA	NA	NA	38
CVD-SLG	Planar diode (Si)	1,550	PB (Plasmonic)	604 (1.9, 0.386)	78	15 (NRZ)	NA	39
CVD-SLG	Planar diode (Si)	1,550	PC	104 (–0.8, 2)	70	128 (NRZ)<br>224 (PAM4)	NA	40
CVD-tBLG	Planar diode (Si)	1,550	PC	648 (0.5, 0.125)	65	25 (OOK)	1.6	This work

Supplementary References

1 Mak, K. F. *et al.* Measurement of the Optical Conductivity of Graphene. *Phys. Rev. Lett.* **101**, 196405 (2008).

2 Chang, Z. & Chiang, K. S. Experimental verification of optical models of graphene with multimode slab waveguides. *Opt. Lett.* **41**, 2129–2132 (2016).

3 Lao, J., Tao, J., Wang, Q. J. & Huang, X. G. Tunable graphene-based plasmonic waveguides: nano modulators and nano attenuators. *Laser Photon. Rev.* **8**, 569–574 (2014).

4 Sorianello, V., Midrio, M. & Romagnoli, M. Design optimization of single and double layer Graphene phase modulators in SOI. *Opt. Exp.* **23**, 6478–6490 (2015).

5 Landgraf, W., Shallcross, S., Türschmann, K., Weckbecker, D. & Pankratov, O. Electronic structure of twisted graphene flakes. *Phy. Rev. B* **87**, 075433 (2013).

6 Yin, J. *et al.* Selectively enhanced photocurrent generation in twisted bilayer graphene with van Hove singularity. *Nat. Commun.* **7**, 10699 (2016).

7 Yu, K. *et al.* Gate tunable optical absorption and band structure of twisted bilayer graphene. *Phy. Rev. B* **99**, 241405 (2019).

8 Tabert, C. J. & Nicol, E. J. Optical conductivity of twisted bilayer graphene. *Phy. Rev. B* **87**, 121402 (2013).

9 Guo, J. *et al.* High-performance silicon−graphene hybrid plasmonic waveguide photodetectors beyond 1.55 μm. *Light. Sci. Appl.* **9**, 29 (2020).

10 Tan, Z. *et al.* Building Large-Domain Twisted Bilayer Graphene with van Hove Singularity. *ACS Nano* **10**, 6725–6730 (2016).

11 Gabor, N. M. *et al.* Hot Carrier–Assisted Intrinsic Photoresponse in Graphene. *Science* **334**, 648–652 (2011).

12 Freitag, M., Low, T., Xia, F. & Avouris, P. Photoconductivity of biased graphene. *Nat. Photon.* **7**, 53–59 (2013).

13 Koppens, F. H. L. *et al.* Photodetectors based on graphene, other two-dimensional materials and hybrid systems. *Nat. Nanotechnol.* **9**, 780–793 (2014).

14 Graham, M. W., Shi, S.-F., Ralph, D. C., Park, J. & McEuen, P. L. Photocurrent measurements of supercollision cooling in graphene. *Nat. Phys.* **9**, 103–108 (2013).

15 Ma, P. *et al.* Plasmonically Enhanced Graphene Photodetector Featuring 100 Gbit/s Data Reception, High Responsivity, and Compact Size. *ACS Photon.* **6**, 154–161 (2019).

16 Schuler, S. *et al.* High-responsivity graphene photodetectors integrated on silicon microring resonators. *Nat. Commun.* **12**, 3733 (2021).

17 Tielrooij, K. J. *et al.* Generation of photovoltage in graphene on a femtosecond timescale through efficient carrier heating. *Nat. Nanotechnol.* **10**, 437–443 (2015).

18 Kim, K. *et al.* Raman Spectroscopy Study of Rotated Double-Layer Graphene: Misorientation-Angle Dependence of Electronic Structure. *Phys. Rev. Lett.* **108**, 246103 (2012).

19 Gan, X. *et al.* Chip-integrated ultrafast graphene photodetector with high responsivity. *Nat. Photon.* **7**, 883–887 (2013).

20 Pospischil, A. *et al.* CMOS-compatible graphene photodetector covering all optical communication bands. *Nat. Photon.* **7**, 892–896 (2013).

21 Wang, X., Cheng, Z., Xu, K., Tsang, H. K. & Xu, J.-B. High-responsivity graphene/silicon-heterostructure waveguide photodetectors. *Nat. Photon.* **7**, 888–891 (2013).

22 Schall, D. *et al.* 50 GBit/s Photodetectors Based on Wafer-Scale Graphene for Integrated Silicon

Photonic Communication Systems. *ACS Photon.* **1**, 781–784 (2014).
23 Youngblood, N., Anugrah, Y., Ma, R., Koester, S. J. & Li, M. Multifunctional Graphene Optical Modulator and Photodetector Integrated on Silicon Waveguides. *Nano. Lett.* **14**, 2741–2746 (2014).
24 Shiue, R.-J. *et al.* High-Responsivity Graphene–Boron Nitride Photodetector and Autocorrelator in a Silicon Photonic Integrated Circuit. *Nano. Lett.* **15**, 7288–7293 (2015).
25 Goykhman, I. *et al.* On-Chip Integrated, Silicon–Graphene Plasmonic Schottky Photodetector with High Responsivity and Avalanche Photogain. *Nano. Lett.* **16**, 3005–3013 (2016).
26 Schuler, S. *et al.* Controlled Generation of a p–n Junction in a Waveguide Integrated Graphene Photodetector. *Nano. Lett.* **16**, 7107–7112 (2016).
27 Schall, D., Porschatis, C., Otto, M. & Neumaier, D. Graphene photodetectors with a bandwidth >76 GHz fabricated in a 6″ wafer process line. *J. Phys. D Appl. Phys.* **50**, 124004 (2017).
28 Gao, Y., Zhou, G., Zhao, N., Tsang, H. K. & Shu, C. High-performance chemical vapor deposited graphene-on-silicon nitride waveguide photodetectors. *Opt. Lett.* **43**, 1399–1402 (2018).
29 Schuler, S. *et al.* Graphene Photodetector Integrated on a Photonic Crystal Defect Waveguide. *ACS Photon.* **5**, 4758–4763 (2018).
30 Muench, J. E. *et al.* Waveguide-Integrated, Plasmonic Enhanced Graphene Photodetectors. *Nano. Lett.* **19**, 7632–7644 (2019).
31 Ding, Y. *et al.* Ultra-compact integrated graphene plasmonic photodetector with bandwidth above 110 GHz. *Nanophotonics* **9**, 317–325 (2020).
32 Flöry, N. *et al.* Waveguide-integrated van der Waals heterostructure photodetector at telecom wavelengths with high speed and high responsivity. *Nat. Nanotechnol.* **15**, 118–124 (2020).
33 Li, J., Yin, Y., Guo, J., Liu, C. & Dai, D. Hybrid ultrathin-silicon/graphene waveguide photodetector with a loop mirror reflector. *Opt. Exp.* **28**, 10726–10736 (2020).
34 Mišeikis, V. *et al.* Ultrafast, Zero-Bias, Graphene Photodetectors with Polymeric Gate Dielectric on Passive Photonic Waveguides. *ACS Nano* **14**, 11190–11204 (2020).
35 Marconi, S. *et al.* Photo thermal effect graphene detector featuring 105 Gbit s−1 NRZ and 120 Gbit s−1 PAM4 direct detection. *Nat. Commun.* **12**, 806 (2021).
36 Wang, Y. *et al.* Ultrahigh-speed graphene-based optical coherent receiver. *Nat. Commun.* **12**, 5076 (2021).
37 Guo, J. *et al.* High-Speed Graphene–Silicon–Graphene Waveguide PDs with High Photo-to-Dark-Current Ratio and Large Linear Dynamic Range. *Laser Photon. Rev.* **17**, 2200555 (2023).
38 Wang, J. *et al.* High-responsivity graphene-on-silicon slot waveguide photodetectors. *Nanoscale* **8**, 13206–13211 (2016).
39 Yan, S., Zuo, Y., Xiao, S., Oxenløwe, L. K. & Ding, Y. Graphene photodetector employing double slot structure with enhanced responsivity and large bandwidth. *Opto-Electron, Adv.* **5**, 210159–210159 (2022).
40 Wang, Y. *et al.* Ultra-Compact High-Speed Polarization Division Multiplexing Optical Receiving Chip Enabled by Graphene-on-Plasmonic Slot Waveguide Photodetectors. *Adv. Opt. Mater.* **9**, 2001215 (2021).
